# Identification of inhibitors of *Plasmodium falciparum* gametocyte development

**DOI:** 10.1186/1475-2875-12-408

**Published:** 2013-11-11

**Authors:** Sandra Duffy, Vicky M Avery

**Affiliations:** 1 Discovery Biology, Eskitis Institute for Drug Discovery, Griffith University, Nathan, Queensland 4111, Australia

**Keywords:** Malaria, High throughput screening, *Plasmodium falciparum*, Gametocytocidal, Drug discovery, Transmission blocking

## Abstract

**Background:**

*Plasmodium falciparum* gametocytes, specifically mature stages, are the only stage in man transmissible to the mosquito vector responsible for malaria transmission. Anti-malarial drugs capable of killing these forms are considered essential for the eradication of malaria. The comprehensive profiling of *in vitro* activity of anti-malarial compounds against both early (I-III) and late (IV-V) stage *P. falciparum* gametocytes, along with the high throughput screening (HTS) outcomes from the MMV malaria box are described.

**Method:**

Two anti-gametocyte HTS assays based on confocal fluorescence microscopy, utilizing both a gametocyte specific protein (pfs16-Luc-GFP) and a viability marker (MitoTracker Red CM-H_2_XRos) (MTR), were used for the measurement of anti-gametocytocidal activity. This combination provided a direct observation of gametocyte number per assay well, whilst defining the viability of each gametocyte imaged.

**Results:**

IC_50_ values were obtained for 36 current anti-malarial compounds for activities against asexual, early and late stage gametocytes. The MMV malaria box was screened and actives progressed for IC_50_ evaluation. Seven % of the “drug-like” and 21% of the “probe-like” compounds from the MMV malaria box demonstrated equivalent activity against both asexual and late stage gametocytes.

**Conclusions:**

The assays described were shown to selectively identify compounds with gametocytocidal activity and have been demonstrated suitable for HTS with the capability of screening in the order of 20,000 compounds per screening campaign, two to three times per seven-day week.

## Background

Malaria is a disease resulting from infection by the intracellular parasite *Plasmodium*. It remains the most significant parasitic disease in the tropics where it causes ~200 million clinical cases and is reported to claim up to 1.2 million lives each year [1]. Substantial efforts are being made to not only reduce the number of clinical manifestations and deaths attributed to malaria, but also to the complete eradication of this disease. The World Health Organization (WHO) first attempted a global eradication programme during the period 1955–1969, which was ultimately unsuccessful. A recent review of the 1955–1969 campaign points to the lack of suitability of the control measures taken at that time for all areas involved [2]. Some countries were highly successful in achieving eradication, whereas in others any headway made was lost to subsequent resurgence of malaria, often in epidemic proportions. International aid for continued malaria control in endemic malaria regions declined considerably during the 1970’s and 1980’s, however recent renewed international efforts into malaria control and research has resulted in promising advances in new control measures.

Artemisinin combination therapy (ACT) has proven to be the most effective anti-malarial for both treatment and control to date, with the additional benefit of significantly impacting on the reduction in malaria transmission [3-5]. In 2007, a renewed effort for global eradication of malaria was proposed by the Bill and Melinda Gates Foundation (BMGF) at the 2007 Malaria Forum in Seattle, Washington [6]. In 2011, scientists reconvened in Bethesda, to discuss the latest research and developments into the sexual development of *Plasmodium* and the latest strategies for malarial control. One of the key deliverables identified from this meeting was the need for phenotypic assays for the identification of compounds which kill gametocytes and ookinetes, and hence may ultimately function as transmission blocking drugs [7].

Gametocytes arise through a switch from asexual replication in the human host to sexual forms of the parasite in a process termed gametocytogenesis. During intra-erythrocytic asexual replication, the stage of the parasite life cycle responsible for the clinical symptoms of malaria, limited numbers of the parasite undergo conversion to the sexual form. This sexual differentiation appears to be triggered and, or controlled by a number of environmental, parasite and host defined mechanisms which are still not clearly defined. Several of these putative gametocytogenesis inducing factors have been recently reviewed by Lucantoni & Avery [8].

Gametocytes remain in human host erythrocytes throughout this conversion and during the prolonged 10–12 day maturation period. Initially, gametocytes are difficult to differentiate from asexual ring and early trophozoite stages by standard Giemsa-stained blood smears. The gametocytes undergo further development, which is classified into five distinct morphological stages, termed I to V. The gametocytes continue a morphological and biochemical development from an early stage or immature (I-III) to a late stage or mature (IV and V) gametocyte. Only the fully developed, mature stage V gametocytes, can survive in the mosquito gut environment where the male and female gametes generated undergo fertilization with the formation of ookinetes, perpetuating the transmission cycle from human host through mosquito vector, and back to the human host.

A number of large-scale high throughput screening (HTS) campaigns have been performed over the last five years to identify compounds with activity against the asexual blood stages of the malaria parasite, *Plasmodium falciparum*. Notably, 309,474 compounds by St. Jude Children’s Research Hospital [9], 2 million by GlaxoSmithKline Tres Cantos [10] and 1.7 million by Novartis-GNF (Genomics Institute of the Novartis Research Foundation) [11] were screened. The not-for-profit organization, Medicines for Malaria Venture (MMV) has also been instrumental in obtaining numerous proprietary chemical libraries from both the pharmaceutical and biotech industries for testing for anti-malarial activity. The screening of these has been undertaken predominantly as a joint venture between MMV and the Eskitis Institute for Drug Discovery, at Griffith University. The collective outcomes of these screening campaigns have resulted in the public availability of data for numerous sets of compounds with asexual anti-malarial activity. The total number of asexual active compounds identified through the aforementioned activities exceeds 25,000. These asexually active compounds have formed the basis of screening activities to identify molecules with dual activity against both the asexual and sexual stages, in a bid to identify molecules with the capability of not only treating the clinical symptoms, but also with the added ability to block transmission.

In order to identify dual acting compounds against asexual and gametocyte stages, we have developed a large-scale, high-yielding, reproducible gametocyte induction protocol, which provides sufficient gametocytes in the quantity and quality required to develop HTS assays for both early (I-III) and late (IV-V) stage gametocytes. Both assays, utilize high throughput confocal microscopy, with automated image analysis for detection and quantification of compound activity against gametocytes. To validate and demonstrate the capability of these assays to identify compounds with activity against early and late stage gametocytes, 36 compounds used as asexual anti-malarials, in addition to compounds currently in preclinical development were tested, and their IC_50_ values against both early and late stage gametocytes determined. The activity of these compounds against the asexual forms of the parasite in an image-based assay was also confirmed [12]. The MMV malaria box was subsequently screened for activity against both early and late stage gametocytes in HTS format and active compounds further characterized by determining their IC_50_ values. The MMV malaria box [13,14] is comprised of compounds which were identified as asexual actives from the Novartis-GNF, St Jude Children’s research hospital, and the GSK-Tres Cantos (TCAMS) active compound sets, as well as from the screening of commercial libraries. Two hundred compounds within the MMV malaria box are considered to be drug-like and 200 probe-like, which may be considered as a representative subset of the 25,000 asexual active compounds identified over *the last 5 years.*

Herein, the first ever comprehensive published data for anti-malarial compounds with activity against both early (I-III) and late (IV-V) stage gametocytes is described. The same gametocyte strain, induction protocol and assay technology has been utilized to provide this data enabling a direct comparison of the results obtained. Also included is the first gametocyte specific data presented for the MMV malaria box with a complete data set in IC_50_ format for activity against both early and late stage gametocytes.

## Methods

### Asexual culture

Asexual culture was performed using standard culturing techniques as first described by Trager and Jensen [15]. Specifically, the asexual culture media contained RPMI with 10 mM Hepes (Invitrogen, Australia), 50 μg/ml hypoxanthine, 2 μg/ml blasticidin (for transgenic selection of NF54-pfs16-GFP), 5% pooled human AB male serum (Sigma, Australia) and 2.5 mg/ml AlbuMAX II® (Invitrogen, Australia). The parasites were maintained at 0.5-4% parasitaemia (%P) at 5% haematocrit (%H). Human O + erythrocytes were obtained from the Australian Red Cross Blood Service (Agreement No: 09-05QLD-06). Human ethics approval was given by the Griffith University Human Ethics Committee for the handling and use of human red blood cells for parasite culture and experiments performed within this study (ESK/03/12/HREC and ESK/03/07/HREC). The culture was adjusted daily to maintain low parasitaemia. All parasite incubations for both culture maintenance and assay were performed at 37°C, 5% CO_2_, 5% O_2_ and 60% humidity.

### *Plasmodium falciparum* 3D7 asexual assay

The asexual parasite culture and assay was performed as described previously [12] with only the single modification of using 5% serum and 2.5 mg/ml AlbuMAX II® in combination rather than 5 mg/ml AlbuMAX II® without serum. In brief, strain 3D7 of *P. falciparum* was synchronized to early trophozoite (‘ring’) stage parasites using sorbitol selective lysis as described previously [16]. On the day of the assay compounds were diluted in water and 5 μl added to PerkinElmer CellCarrier imaging plates. Twenty-five μl of asexual assay media was added to all wells followed by 20 μl of parasite culture at 2% P and 0.75% H, resulting in a final %H of 0.3%. Plates were incubated for 72 hours in standard conditions then stained with 4′,6-diamidino-2-phenylindole (DAPI). The plates were left for at least 4 hours before imaging on the Opera QEHS microplate confocal imaging system (PerkinElmer, Australia). DNA spot analysis was performed and the number of infected red blood cells (iRBCs) provided as the output. The OPERA output was defined as the number of classified spots per imaged area and was calculated as % inhibition based on 2 μM artemisinin (positive) and 0.4% DMSO (negative) controls. IC_50_ values were determined using Prizm 4.

### Gametocyte induction, culture and isolation

NF54 transgenic parasite expressing GFP linked to luciferase under the control of the early gametocyte specific pfs16 promoter, Pfs16 [17] were generously provided by Prof David Fidock, Columbia University, NY and maintained in asexual culture as described above. At Day −3 of the induction protocol, mid stage trophozoite parasites were isolated on a CS magnetic column (MACS) and VarioMACS separator (Miltenyi Biotec, Australia). Fresh non-infected red blood cells (RBCs) were added to the isolated trophozoites, and the haematocrit reduced to 1.25%. After overnight shaking, the Day −2 culture was then put under nutritional stress overnight. Resulting trophozoite parasites, Day −1, were then adjusted to 3% P and shaken overnight before removal of spontaneously generated gametocytes using MACS column gametocyte depletion on Day 0. Ring stage parasites were then transferred into gametocyte culture media which was the same as the asexual media with the addition of 50 mM N-acetylglucosamine (NAG) for removal of asexual parasites.

### Post induction, culture and isolation of gametocytes for the early (I-III) stage assay

Day 0 ring stage parasites, post spontaneous gametocyte removal, were centrifuged and suspended in asexual media. The ring stage parasites were split to 4% P and the %H maintained at 2.5%. The culture was left overnight to incubate. The following day (Day1 post-induction) fresh media was added to the mid trophozoite stage culture by simple aspiration and replacement and the culture incubated overnight. After overnight incubation the non-committed asexual parasites were again at ring stage, however, the committed gametocytes were now similar to young trophozoite stages and contained haemozoin. The gametocytes were isolated using the MAC isolation procedure. Twenty μl of non-infected compacted RBCs were added per isolated gametocyte column run (10 ml/run) resulting in a final haematocrit of between 0.2 and 0.5% H. Determination of % gametocytes was made by both Giemsa-staining and GFP fluorescence microscopy. Gametocytes isolated in this manner were typically stage I/IIa. In preparation for use in the early stage assay, these I/IIa gametocytes were diluted to 10%P in 0.1% H in media containing NAG.

### Post induction culture and isolation of gametocytes for the late (IV-V) stage assay

The Day 0 ring parasites were placed in media containing 50 mM NAG at 2.5% H, and 50 ml of culture was placed in 100 mm diameter large Petri dishes. Thirty-five ml of spent media was replaced daily over 7 successive days for each culture dish. On Day 8 the gametocytes were harvested using magnetic columns and RBCs added to the isolated gametocytes. The final isolated gametocytes and RBCs were then counted on a haemocytometer, and the culture adjusted to 10% gametocytes in a total of 0.1% H in media containing 50 mM NAG.

### Early gametocyte (stage I-III) assay protocol

Five μl of diluted compound in 4% DMSO was transferred in 384-well format into PerkinElmer Cell carrier Poly-D-Lysine imaging plates using a Minitrack™ liquid handler (CCS Packard). The day 2 isolated gametocytes prepared at 10% P and 0.1% H were dispensed in 45 μl volumes into the imaging plates using a multidrop dispensing instrument (Thermo Scientific, Australia). The plates were sealed with gas permeable membranes (BreathEasy, BEM-1, Diversified Biotech) and incubated for 72 hours in standard incubation conditions of 5% CO_2_, 5% O_2_ and 60% humidity at 37°C. After 72 hours incubation, 5 μl of 0.07 μg/ml MitoTracker Red CM-H_2_XRos (MTR) (Invitrogen, Australia) in phosphate buffered saline (PBS) was added to each well, and plates were resealed with membranes and incubated overnight under standard conditions. The following day, the plates were brought to room temperature for at least one hour before being measured on the Opera QEHS Instrument (PerkinElmer), utilizing a Twister II micro-plate handler (Perkin Elmer) for automated plate feeding to allow for “walk away” measurement and image analysis.

### Late Gametocyte assay protocol (stage IV-V))

Five μl of diluted compound in 4% DMSO were transferred in 384-well format into PerkinElmer Cell carrier PDL imaging plates using a Minitrack™ liquid handler. The day 8 isolated gametocytes prepared at 10% P and 0.1% H were dispensed in 45 μl volumes into the imaging plates using a multidrop dispensing instrument. The plates were sealed with gas permeable membranes (4ti-05 15/ST, 4titude Surry, UK,) and incubated for 72 hours in standard incubation conditions. After 72 hours incubation, 5 μl of 0.07 μg/ml MTR in PBS were added per well, and plates resealed with membranes and incubated overnight under standard conditions. The following day, the plates were brought to room temperature for at least one hour before being measured on the Opera QEHS Instrument (PerkinElmer), utilizing a Twister II microplate handler (Perkin Elmer) for automated plate feeding to allow for “walk away” measurement and image analysis.

### Opera image acquisition and analysis

Image acquisition and analysis for both the early and late stage gametocyte assays was undertaken on the Opera QEHS micro-plate confocal imaging system (PerkinElmer).

**Figure 1:**
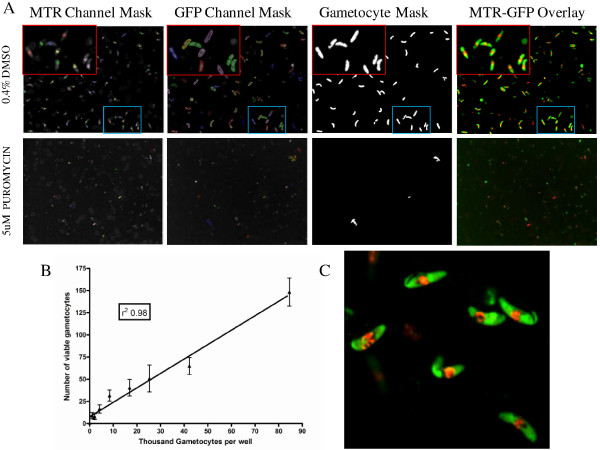
**Script analysis and performance of late stage gametocyte assay. A**. Top panel no compound treatment. Bottom panel 5 μM puromycin, 72 hour treated late stage gametocytes. MTR channel mask, GFP channel Mask, analysed Gametocyte Mask and MTR-GFP overlay of Opera acquired images. **B**. Linearity graph- gametocyte number per well against script identified viable gametocytes per well. **C**. Opera acquired image of gametocytes after 72 hours incubation and MTR staining. A X60 water objective was used to capture detailed images. Classical stage V gametocytes are observed.

Single images were taken for each well at 3 μm from the bottom of the PerkinElmer CellCarrier plate using a 20X water immersion objective. First, the GFP intensity was measured and distribution determined with an exposure time of 400 msec (488 nm), then to ascertain the fluorescence intensity of the viability stain, MTR, measurements were made for 600 msec (532 nm). Autofocus from the first well was used to correct for the subsequent well images. All images, for each well, were analysed by the Acapella based script and stored directly into a database as imaged. The script has been optimized to select objects with a MTR fluorescent signal above an assay specified cut-off and a GFP object morphology which was longer than it was wider, specifically the object was elongated (Figure 1A). The number of elongated objects was equivalent to the number of gametocytes per area of the well imaged (Figure 1B). The late stage gametocytes, after 72 hours incubation and MTR staining, have a classical stage V morphology as can be seen from the GFP-MTR image acquired on the Opera QEHS using 60× magnification (Figure 1C) demonstrating the continued development path of the gametocytes from stage IV to stage V within the 384-well micro-titer imaging plates.

### Demonstration of linearity between parasite number per well and script detected number of parasites per imaged area

Day 8 Gametocytes (stage IV) isolated by MACs column were counted for total number of cells and the number of gametocyte infected cells. Dilutions were made to the appropriate number of gametocytes/ml in a final total haematocrit of 0.1%. It was determined that 75 million RBCs per ml was equivalent to 1% H, thus allowing for rapid translation from gametocyte number and RBCs/ml to %P and %H values [12]. Individual cultures containing the range of gametocyte numbers per well (1,000-85,000) were dispensed into 16 wells per cell density and the plates incubated for 72 hours. After incubation, 5 μl of MTR (prepared as described above) was added to all wells and the plates incubated overnight in standard conditions. The assay plate was then imaged at standard exposures and the script applied to the images as described above. The number of identified gametocytes per well image (average ± SD) was plotted against the actual number of gametocytes per well using linear regression in Graph pad Prizm (Figure 1B).

### Compound preparation

#### Solid anti-malarial compound stock preparations for IC_50_ determination

The appropriate volume of 100% DMSO was added to the vials of solid anti-malarial compounds to give 10 mM final stock concentrates. The compounds were diluted in 384-well Eppendorf deep well storage block plates in duplicate point across the plate. The dilutions made resulted in three concentrations per log dose for each compound tested. All the dilutions were made in 100% DMSO. For the assays, 1 μl of compound was pre-diluted into 24 μl of sterile water before addition to the assay plate to maintain a final DMSO concentration of 0.4%. Samples were tested on the 3D7 asexual, early and late stage gametocyte assays.

#### MMV malaria box compound handling

The MMV malaria box compounds were received as 10 mM stocks in 100% DMSO. To maximize the small volume available, the compounds were further diluted to a final stock concentration of 2.5 mM in 100% DMSO and replicate sets of plates made for future use. These stocks were stored at −20°C and once thawed were not repeat freeze thawed in order to maintain the integrity of the compounds. For initial testing, the compounds were used as either 1/2 or 1/10 dilution in duplicate point to determine % inhibition at 5 μM and 0.5 μM compound concentrations. DMSO stock compounds were diluted 1/25 in water for use in the assays to ensure a DMSO concentration of less than 0.5%.

Compounds demonstrating >50% inhibition on the late stage assay at 0.5 μM or >80% at 5 μM were taken for determination of accurate IC_50_ values against both the early and late stage gametocyte assays in duplicate point. Early stage actives at >50% at 0.5 μM were also taken for IC_50_ evaluation against both early and late stage gametocytes. Compounds demonstrating only low activity against early stage gametocytes were de-prioritized at this point and not taken further for retest.

#### Opera data analysis

The Opera script output describes the number of viable gametocytes identified. The reduction in the number of gametocytes obtained after compound addition and 72 hours incubation was calculated as a percentage of the positive and negative controls contained in each assay plate. The positive control constitutes 16 wells of 5 μM puromycin and the negative control was 16 wells of 0.4% DMSO. The individual ‘in-plate’ controls were used to calculate the relative (R) % inhibition of parasite number for the unknown wells. The equation used is as below.

$$\begin{array}{l} R\;\%\;\mathrm{inhibition}=100‒\left(\mathrm{Unknown}‒5\mathrm{\mu M}\;\mathrm{puromycin}\right)/\\ \;\left(0.4\%\mathrm{DMSO}‒5\mathrm{\mu M}\;\mathrm{puromycin}\right)\times 100 \end{array}$$

The relative 100% inhibition control of 5 μM puromycin was used in all calculation as this is relevant for comparing the action of many unknown compounds, specifically on the parasite development and viability than the use of absolute zero in the form of non-infected RBC’s.

#### IC_50_ calculation

Normalised % inhibition of parasite numbers were plotted against log concentration (conc) of the compound using a 4 parameter log dose, non-linear regression analysis, with sigmoidal dose response (variable slope) curve fit using Prizm 4.0. No constraints were placed on the top, bottom or Hill slope of the curve fit in the graphing software. Compounds which did not reach a maximal inhibition plateau could not, therefore, have IC_50_ values determined by this analysis.

## Results

### High-throughput confocal imaging and assay linearity (Late stage IV-V)

Gametocytes were automatically classified and counted using an Opera QEHS high throughput confocal imager. Image analysis script parameters were selected to determine the number of viable late stage gametocytes in the imaged area. A linear relationship was observed between the numbers of gametocytes per well determined using a haemocytometer compared to gametocyte objects automatically calculated (Figure 1B). An r^2^ value of 0.98 was obtained and linearity was maintained from 1,000 to 85,000 gametocytes per well.

### Current anti-malarial compounds

The IC_50_ data generated for known anti-malarials against early and late stage gametocytes, as well as asexual 3D7 parasites, is presented in duplicate point for 21 concentrations in Table 1. Compounds were tested at the maximum concentration of 40 μM, thus compounds which did not yield an IC_50_ value, within the confines of the analysis parameters, were either expressed as the % inhibition at 40 μM or not active (NA) at 40 μM. The selectivity index (SI), a ratio of compound activity on asexual forms compared to activity in either early or late gametocytes, an important parameter for determining dual activity, is also shown in Table 1.

**Table 1:** Activity data for anti-malarial drugs

**COMPOUND**	**3D7**^***a***^	**NF54-pfs16-GFP**^***a***^	**NF54-pfs16-GFP**^***b***^	**(I-III)/ABS**	**(IV-V)/ABS**
	**ABS**	**early (I-III) gam**	**late (IV-V) gam**		
**4-Aminoquinolines**					
Amodiaquine	255 (65)	242 (110)	87%	0.9	
Naphthoquine^s^	411 (82)	483 (330)	NA	1.2	
AQ-13	183 (0.01)	453 (230)	82%	2.5	
Piperaquine^s^	1880 (210)	3990 (2800)	43%	1.6	
Hydroxychloroquine^s^	966 (130)	1340 (140)	45%	1.4	
Pyronaridine	299 (69)	250 (96)	4260 (240)	0.8	14.2
**8-Aminoquinolines**					
Pamaquine	88%	79%	NA		
Primaquine	7120 (1500)	NA	74%		
Tafenoquine	5650 (1600)	4800 (1500)	4620 (1600)	0.8	0.8
NPC-1161B	3360 (860)	2180*	4850 (2700)	0.6	1.4
**Endoperoxides**					
Artemether	25.8 (11)	11 (2.8)	3.13 (0.7)	0.4	0.1
Artenimol (DHA)	2.08 (0.6)	18.3 (17)	2.17 (1.2)	8.8	1.0
Artesunate	6.7 (1.6)	3.8 (0.28)	2.53 (1.10)	0.6	0.4
Artemisinin	41.6 (12)	12.1 (14)	5.47 (2.0)	0.3	0.1
Artemisone	3.72 (0.14)	2.4 (0.85)	1.7 (0.46)	0.6	0.5
**Anti folates**					
Pyrimethamine	21.5 (0.07)	NA	NA		
Chlorproguanil	6250 (520)	108%	4340 (840)		0.7
Proguanil HCl	78%	6540*	84%		
Dapsone	NA	NA	NA		
**Sulphonamides**					
Sulfadiazine	NA	NA	NA		
Sulfamethoxazole	NA	NA	NA		
Sulfadoxine	NA	NA	NA		
**Amino alcohols**					
Halofantrine	136 (66)	135 (98)	31.9 (11)	1.0	0.2
Lumefantrine^s^	137 (70)	107 (137)	2.07 (0.55)	0.8	0.0
Mefloquine (Racemic)	158 (33)	223 (120)	100 (3.6)	1.4	0.6
Mefloquine (+ RS)	133 (41)	226 (76)	134 (110)	1.7	1.0
Quinine sulfate	217 (0.7)	2110 (280)	318 (180)	9.7	1.5
**Antibiotics**					
Trimethoprim	3740 (120)	NA	NA		
Thiostrepton	913 (210)	959 (570)	556 (58)	1.1	0.6
Clindamycin	325 (64)	NA	NA		
Cis-mirincamycin (HCl)	NA	NA	64%		
Trans-mirincamycin (HCl)	86%	NA	66%		
Tetracycline	2270 (170)	NA	NA		
**Other**					
Fosmidomycin mono sodium^s^	91%	NA	NA		
Methylene blue trihydrate	21.9 (0.07)	280 (130)	287 (100)	12.8	13.1
Pentamidine	697 (36)	1560 (620)	2850 (540)	2.2	4.1

*ABS* Asexual blood stage, *gam* gametocyte, ^*a*^n = 2, ^*b*^n = 3. IC_50_ nM (SD), * n = 1 only, *NA* Not active at 40 μM, % at 40 μM. ^s^Potential solubility issues with stock compounds in DMSO.

### 4-Aminoquinolines

Amodiaquine (AQ), naphthoquine (NQ), AQ-13, piperaquine (PIPQ), hydroxychloroquine (HQ) and pyronaridine (PYRO) all exhibited comparable activity against both asexual 3D7 parasites and early stage gametocytes (**AQ** 255:242, **NQ** 411:483, **AQ-13** 183:453, **PIPQ**1880: 3990, **HQ** 966: 1340, **PYRO** 299: 250 nM, 3D7 asexual: early gametocytes, respectively). However, except for PYRO, no activity against late stage gametocytes was observed for any of the other compounds tested up to concentrations of 7 μM. PRYO had an average IC_50_ value of 4260 ± 240 nM (SD) against late stage gametocytes, and was demonstrated to be 14 times more selective for the asexual parasite than late stage gametocytes.

### 8-Aminoquinolines

Pamaquine (PAMQ) demonstrated no significant activity against 3D7 asexual, early or late stage gametocytes. Primaquine (PQ) exhibited only low levels of activity (7.12 μM ± 1500 (SD)) against the asexual forms in the 3D7 asexual assay, and no significant activity against either early or late stage gametocytes. Tafenoquine (TQ) and NPC-1161B demonstrated equal but low μM activity against the asexual, early and late stage gametocytes.

(**TQ** 5.6: 4.8: 4.6 uM, **NPC-1161B** 3.4:2.2:4.9 μM 3D7 asexual: early gametocytes: late gametocytes respectively).

### Endoperoxides

Artemether, dihydroartemisinin (DHA), artesunate, artemisinin and artemisone all demonstrated similar activity between the 3D7 asexual parasite, early gametocytes and late gametocytes, with IC_50_ values less than 50 nM.

### Antifolates

Of the five antifolates tested, chlorproguanil had similar activity against the 3D7 asexual parasites and late stage gametocytes, with low activity (108% inhibition at 40 μM) against the early gametocytes. Pyrimethamine demonstrated a high level of activity against the 3D7 asexual parasite, with an IC_50_ value of 21.5, ± 0.07 nM (SD) but had no activity against either the early or late stage gametocytes.

### Sulphonamides

Of the three compounds of this chemical class tested, sulphadiazine, sulphamethoxazole and sulphadoxine, none had activity against asexual or gametocyte stages.

### Amino alcohols

The amino alcohols tested included halofantrine (HF), lumefantrine (LF), mefloquine (MQ) and quinine (Q). All demonstrated equivalent activity against early and late stage gametocytes, apart from Q, which was more active against the late stage gametocytes than early stage gametocytes. (**HF** 136:135:32 nM, **LF** 137:107:2.1 nM, **MQ** (Racemic) 158:223:100 nM, **Q** 217:2110:318 nM asexual: early gametocytes: late gametocytes, respectively).

### Antibiotics

Of the six antibiotics tested, trimethoprim, thiostepton, clindamycin, mirincamycin (cis and trans isomers) and tetracycline, only thiostrepton demonstrated comparable activity against both early and late stage gametocytes (0.5 – 1 μM), as well as the asexual 3D7 parasite.

### Other

Methylene blue and pentamidine were also tested against all stages. Methylene blue was active against both early and late stage gametocytes at less than 300 nM, but was almost 10 fold less active than against the 3D7 asexual parasite. Pentamidine was slightly less active against early and late stage gametocytes (1.56:2.85 μM early: late) in comparison to its 3D7 asexual activity (0.69 μM).

### MMV malaria box HTS

**Figure 2:**
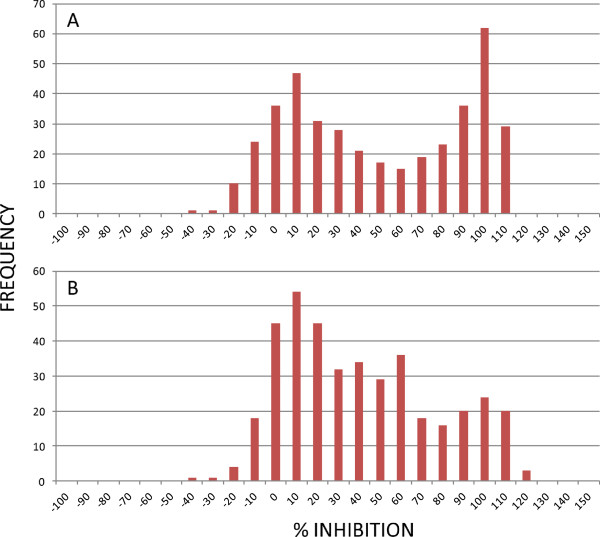
**Compound activity distribution against early (I-III) and late (IV-V) stage gametocytes.** Normal distribution of the activity of MMV malaria box compounds against the early **(A)** and late **(B)** stage gametocytes was not observed as the compound set has an asexual bias.

Duplicate point screening at 5 μM and 0.5 μM doses was performed for the MMV malaria box prepared as described above, on both the early and late stage gametocyte assays. The activity distribution generated for both assays (Figures 2A and B) did not produce a normal distribution; however this was expected due to the library being primarily comprised of compounds with demonstrated asexual anti-malarial activity. It is noted that due to the use of relative % inhibition in relation to 5 μM puromycin some compounds demonstrate a value greater than 100% inhibition. A number of parameters can cause this, including compound related effects such as lysis of all RBCs, specific lysis of iRBCs, or compound fluorescence quenching of the GFP or MTR signal.

**Figure 3:**
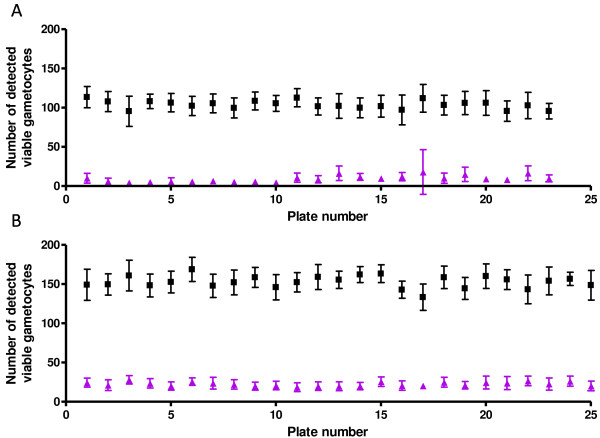
**In plate control data for early (I-III) and late (IV-V) stage gametocyte assays.** In-plate control data obtained during screening of the MMV malaria box against **A**. early (I-III) and **B**. late (IV-V) stage gametocytes is shown. Black squares represent no inhibition (vehicle control: 0.4% DMSO) and purple triangles indicate 100% inhibition (5 μM puromycin).

IC_50_ evaluation was performed on compounds with activity greater than 50% for the 0.5 μM dose and, or greater than 80% at 5 μM, for the late stage gametocyte assay. IC_50_ value determination was performed for 97 compounds on fresh stock concentrates. Both early and late stage gametocyte assays were performed for the evaluation of the 97 early or late stage active compounds from the MMV malaria box. IC_50 value determination_ was performed in a single experiment in duplicate point at 16 concentrations per compound tested. The in-plate controls (comprised of 16 wells of 5 μM puromycin as the positive control and 16 wells of 0.4% DMSO as the negative control) for 25 × 384-well plates tested to obtain accurate IC_50_ values, for early and late stage gametocyte, are presented in Figures 3A and B. The average signal: background values were 13.2 ± 5.5 (SD) and 6.9 ± 0.76 (SD) for early and late, respectively, with Z’ values of 0.68 ± 0.1(SD) and 0.53 ± 0.08 (SD), respectively. The performance of the assays was highly stable from the first to last plates with good reproducibility within a single plate. Unfortunately, IC_50_ values for 25 of these compounds could not be determined for either early and/or late stage assays due to low activity or an inability to generate suitable curves. Many of the compounds were also shown to be insoluble in water and thus the necessary dilution of DMSO for performing the assay was limiting.

**Figure 4:**
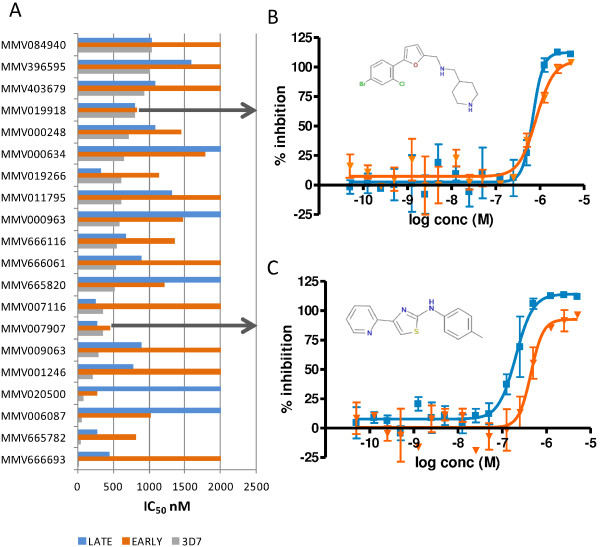
**Early and late stage gametocytocidal activity of MMV Malaria Box “drug-like” compounds aligned with 3D7 asexual activity. A**. Activity of compounds classified as "drug-like". **B**. MMV019918 IC_50_ evaluation. **C**. MMV007907 IC_50_ evaluation. Orange triangles are indicative of the early (I-III) stage gametocyte activity whereas the blue squares represent the late (IV-V) stage gametocyte activity.

Seventy-two compounds had IC_50_ values ≤1600 nM for either early or late stage gametocytes and were in this case classified as actives as an IC_50_ was obtainable from the Prizm calculation. Compound activities were then assessed according to their “drug-like” (20) or “probe-like” (52) properties as defined by MMV [14]. The data for the IC_50_ values (nM) for both the early and late gametocyte assays for the “drug-like” compounds were plotted against the corresponding 3D7 data provided by MMV and originally generated within this laboratory (Figure 4A). Where an IC_50_ value was not obtainable a maximum IC_50_ value of 2,000 nM was assigned to the sample for graphing purposes to indicate not active (NA) at 5,000 nM or activity greater than 2,000 nM.

Only 20 of the 200 compounds classified as ‘drug-like’ (10%) had activity against either early or late stage gametocytes. Of the 20 ‘drug-like’ active compounds, 7 (35%) were active against both early and late stage gametocytes, 9 (45%) were active against late stage gametocytes only and 4 (20%) were classified as active against early gametocytes only. When considering the degree of activity against the asexual parasite for the 200 ‘drug-like’ compounds in comparison to the late stage gametocyte activity, only 14 (7%) had activity with less than fivefold difference in IC_50_ values, indicating equipotent activities between asexual and late gametocyte stages. One compound in particular, MMV666693, although very active against the 3D7 asexual parasite (23.8 nM) was 18.4 times less active against the late stage gametocyte and not active against the early stage gametocytes at all. As late stage gametocyte activity equal to or better than asexual activity is widely considered to be essential for potential transmission blocking drugs, then only 7% (14/200) of the asexual active ‘drug-like’ compounds within the MMV malaria box would be selected for progression on this basis. Of particular note are MMV019918 and MMV07907 which have IC_50_ values <1,000 nM against all three stages tested (Figure 4B and C).

**Figure 5:**
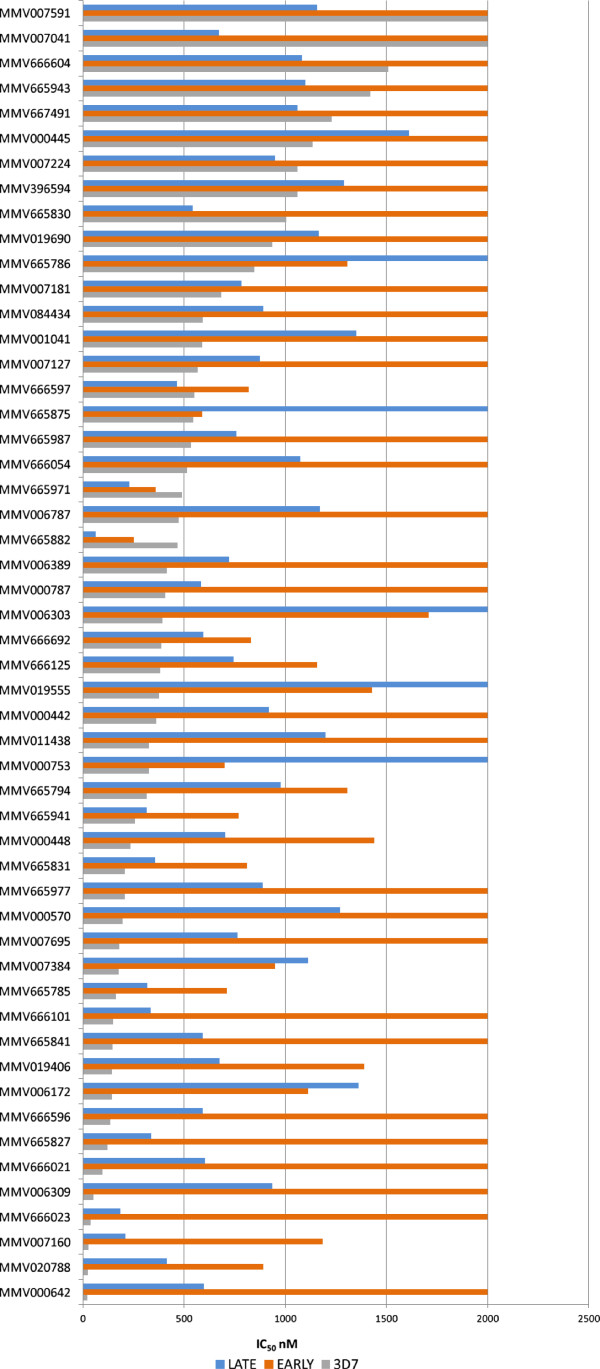
**Early and late stage gametocytocidal activity of MMV Malaria Box “probe-like” compounds aligned with 3D7 asexual activity.** The activities of compounds from the MMV malaria box for the early (I-III) (orange) and late (IV-V) (blue) gametocyte stages are aligned with the activities determined for 3D7 asexual parasites (grey). All three assays were performed using image based assays and analysis.

Of the 200 ‘probe-like’ compounds tested, 52 (26%) gave an IC_50_ value for either early, late or both gametocyte stages ≤1600 nM and were classified as actives (Figure 5). Of these 52 ‘probe-like’ compounds 16 (31%) demonstrated activity against both early and late stage gametocytes, 32 (61%) showed late stage activity only whilst only 5 (9.6%) of the 52 probes exhibited activity for early stage gametocytes only. Of the 200 “probe-like” compounds, 41 (21%) with late stage activity had less than a five-fold reduced activity against late stage gametocytes in comparison to the asexual activity. This is significantly higher than that for “drug- like” compounds (7%). It would, therefore, appear that the ‘probe-like’ compounds within the collection have a higher hit-rate on these gametocyte assays than the ‘drug-like’ compounds (26% “probe-like” late stage gametocyte active in comparison to 10% “drug-like” late stage gametocyte active), Additionally, in general the more “probe-like” compounds have comparable asexual and late stage gametocyte activity than the “drug-like” compounds. The HTS screening data for the MMV malaria box at 5 μM and the IC_50_ values obtained (nM) are incorporated in Additional file 1.

## Discussion

To perform the gametocyte screening assays it was essential to not only have healthy gametocytes in sufficient quantities, but also to be capable of producing highly reproducible quantities according to the required HTS schedule. With the specifically optimized synchronous gametocyte induction and isolation protocol described above, quantities of gametocytes sufficient for 50 × 384-well imaging plates (almost 20,000 data points) were able to be produced two to three times per week. The assays described here were able to detect compounds which prevented the development of gametocytes from stage I to stage III or kill stage IV/V gametocytes.

The linearity calibration conducted during the validation of this assay showed a linear relationship between actual number of gametocytes added per well, as defined by the use of a haemocytometer, and those gametocytes identified per imaged area within a well using the automated image analysis algorithm. Using 33,000 gametocytes per well (384-well plate) in a HTS assay format, an average signal to background (S:B) ratio of 13.2 ± 5.5, 6.9 ± 0.76 (SD) and a Z’ of 0.68 ± 0.1, 0.53 ± 0.08 (SD) for the early and late stage gametocyte assays, respectively were obtained. This data was generated for a screening run of 25 assay plates in 384-well formats, which translates to 9,600 data points.

Several laboratories have developed assays to measure compound activity against late stage gametocytes. Unfortunately, it is difficult to directly compare these assays and associated compound activities with one another as the same compounds have not been screened in all cases. In some instances IC_50_ values have been determined, whereas in others only the % inhibitions at a specific single concentration have been indicated. In addition, the various assays employ different gametocyte induction protocols and parasite strains, isolation protocols, gametocyte densities per well, the presence or absence of RBCs, compound incubation times and media components, as well as methods for detection, which may all have some effect on the results obtained. It is therefore only possible to perform a limited comparison between the compound activities determined by the different assays. Thus, the interpretation of the observed differences and similarities, and resultant implications must be treated with caution. For example two independent laboratories [18,19] have developed late stage gametocyte assays, both using ATP bioluminescence as the measure of parasite viability, and screened some, if not all of the 36 current anti-malarials described here. Although the same thirteen compounds were tested by both groups, one performed IC_50_ values directly whilst the other screened at 10 μM first to select actives. Three compounds have IC_50_ data presented in both publications, which are methylene blue 490 nM and 12 nM, artesunate 10.8 μM and 2.3 μM and pyronaridine 3.2 μM and 0.28 μM. Although the detection method was based on the same technology, incubation times and gametocyte numbers per well were different and may provide the explanation for the variations observed. An assay based on the gametocyte specific NF54-mal8p 1.16- luciferase transgenic parasite [17] has also been use to test activity of a selection of anti-malarial drugs. Activity was expressed in % activity ranges for fixed compound doses related to asexual determined IC_50_ values. DHA was demonstrated to result in 50-75% inhibition of late stage gametocytes at a fixed dose of 120 nM in comparison to less than 50% inhibition for 10 μM or 3.6 μM obtained by ATP bioluminescence [18,19]. Methylene blue exhibited >75% inhibition at 150 nM, which is comparable to the data reported for the ATP Bioluminescence assay [18]. An assay based on lactate dehydrogenase (pLDH) detection, where parasite LDH has higher activity than that of RBCs, was recently reported [20]. This assay is undertaken in two formats, one for 72 hours with the drug in contact with the gametocytes and one where the drug is removed after 72 hours exposure and the plates incubated for a further 72 hours. DHA and methylene blue were both recorded as having activity >400 nM at 72 hours, and 17 and 29 nM, respectively after a further 72 hours of incubation in the absence of drug. This group also tested the non-specific compound, epoxomicin, for which an extended incubation format resulted in an IC_50_ value of 3.9 nM. Epoxomicin was also tested in an ATP bioluminescence assay and gave an IC_50_ value of 0.42 nM [18]. Yet another late stage gametocyte assay using the oxido-reduction indicator Alamar blue has been published which also tested epoxomicin [21] generating an IC_50_ value of 1.4 nM. This compound has been tested in the late stage (IV-V) gametocyte imaging assay described here resulting in an average IC_50_ value of 0.66 ± 0.26 nM over 19 experiments. Notably, this compound, whilst non-specific for the malaria parasite when tested in four different assays, has the most consistent result compared with any other compound tested. Specific anti-malarial compounds have somewhat of a more variable result within the assays reviewed. When comparing the data obtained from the imaging assays described here for known anti-malarial compounds some significant differences and similarities with those presented within the published literature are seen. In general major differences have been observed between results obtained for the endoperoxides.

**Figure 6:**
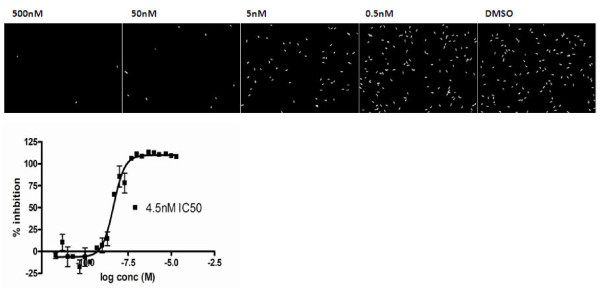
**Images with masked viable elongated gametocytes treated with five concentrations of artesunate.** Representative images of the impact of increasing concentrations of artesunate on late stage (IV-V) gametocytes. Graphic illustration of the activity profile of artesunate against the late stage (IV-V) gametocytes activity.

Although previously it has been widely accepted, or at least predicted, that these compounds are not, or will not be active against late stage gametocytes, in fact all members of this class of compounds result in the death of late stage gametocytes in the assays described here. Artesunate has an average IC_50_ value of 3 nM against late stage gametocytes as determined using the imaging assay. Figure 6 shows the IC_50_ plot for artesunate and the number of viable elongated gametocytes identified decreasing with increasing compound concentration. The parasites within the wells can clearly be seen to have lost MTR staining with complete disruption of gametocyte structure at 50 nM dosing. This is comparable to the effect observed for the GFP-MTR overlay for the 5 μM puromycin control in Figure 1A.

From this very limited literature review of *in vitro* activity against late stage gametocytes in high throughput micro-titre well formats, endoperoxides (namely, DHA or artesunate) are demonstrated to be nM active against late stage gametocytes in one gametocyte specific luciferase reporter assay [17], a pLDH assay [20] plus the late stage gametocyte imaging assay described here. Two assays, both using ATP bioluminescence [18,19], demonstrate low μM activity of endoperoxides against late stage gametocytes. From a literature search, other publications which tested the endoperoxides directly on late stage gametocytes in micro-titre plate HTS assays were not identified. However, other independent laboratories have used endoperoxides as controls for anti-gametocyte activity testing using more traditional Giemsa smears and microscopy, reporting an IC_50_ value of 108 nM for artesunate [22].

The data generated for the endoperoxides within these image based assays clearly show equipotent activity against asexual, early and late stage gametocytes which has previously never been demonstrated. This is a vital piece of information for interpreting field based studies on transmission rates and transmission blockage. Although numerous reports of reduced transmission rates with ACT are presented, the rational for the effect has been predominantly based on the belief that the action is due to the extremely fast clearance of asexual parasites, and action specifically against young sequestered gametocytes. However, the data presented here would indicate that the action could also be against more mature forms of gametocytes released into the circulation.

Some advantages of the image based gametocyte assays are, the same gametocytes, reagents and technology are used for both early and late stage assays and hence the data can be compared directly. The confocal fluorescent microscopy output is a direct measurement of compound effect on individual gametocytes and not the contents of a well in total. The Pfs16-GFP and MTR signal are both specific to the gametocyte as erythrocytes do not express Pfs16 or have mitochondria to convert the MTR viability marker. Hence the host RBC has no direct impact on the assay output.

With this comprehensive evaluation and validation of compound activity against both early and late stage gametocytes for current anti-malarial drugs, a benchmark for comparison of unknown compounds was established. The MMV malaria box, provided a starting point for the search for compounds with activity against the asexual parasite and also against both early and late stage gametocytes. Compounds with activity against both early and late stage gametocytes were equally distributed through the “drug-like” (35%) and “probe-like” (31%) compounds within the MMV malaria box. However, the “probe-like” compounds resulted in a 16% higher hit rate for late stage gametocytes than the “drug-like” compounds, whereas the reverse was true for the early stage gametocytes, with twice the number of the “drug-like” compounds being actives. The “probe-like” compounds also have three times the number of compounds demonstrating asexual and late stage gametocyte activity at less than a five-fold difference and hence, considered to be equipotent against both the asexual blood stage parasites and late stage gametocytes. Of particular note are MMV007907 and MMV019918 which have IC_50_ values < 1000 nM against all three stages tested (Figure 4B and C). These compounds fall into the “drug-like” classification given to the MMV malaria box compounds. Having biological activity against the asexual blood stages, early and late stage gametocytes, in addition to their “drug-like” chemical characteristics have ear-marked these compounds for further investigation.

It could be interpreted from the data that compounds with the classified “drug like” properties are less likely to be equipotent against late stage gametocytes and the asexual blood stage parasites. However, this is a small compound subset and the exact breakdown of the “drug-like” and “probe-like” actives has not been considered in this instance. Based on this data it could be extrapolated that only 14% of the compounds identified as asexually active, irrespective of “drug-like” status, would potentially result in compounds with equipotent activity against asexual blood stage parasites and late stage gametocytes. This is not taking into consideration the activity against early stage gametocytes, which makes the number of total compounds with equipotent activity less.

Hence, 14% of 25,000 asexual actives could potentially yield 3,500 chemical starting points. It must be noted that activity against early or late stage gametocytes does not guarantee that a compound will have transmission-blocking ability in the field or indeed within *in vivo* models. The outcome of these HTS assays is the identification of compounds with activity against gametocytes. With the HTS capacity of the assays described, hundreds of thousands of compounds can be screened for anti-gametocyte activity, enriching the potential compound sets for testing in lower throughput *in vitro* and *in vivo* transmission blocking assays. Essentially, having more chemical starting points to screen in transmission blocking *in vitro* and *in vivo* assays should ultimately increase the odds of finding a compound capable of transmission blockage.

A new lead compound, ELQ-300 (Quinolone-3-diarylether) has demonstrated significant *in vivo* transmission blocking potential in mouse models of transmission [23]. This compound was tested using this late stage gametocyte imaging assay and has an IC_50_ value of 71.9 nM [23] indicating the late stage gametocyte assay as having the ability to identify compounds with transmission blocking potential. The testing of asexual active compounds against gametocytes is an obvious starting point to search for compounds active against gametocytes (25,000 in total), however the alternative approach is to screen whole libraries for compounds active against late stage gametocytes. This late stage gametocyte assay is fully capable of screening compounds in true HTS format, namely the screening of hundreds of thousands of compounds.

## Conclusion

The development of two image based assays for determining activity against early and late stage gametocytes has enabled a comprehensive examination of gametocyte stage specific activity for current anti-malarial drugs and identification of potential new anti-malarial chemical starting points. The same parasite and gametocyte induction protocol, as well as the same technology was used for both early and late assays allowing for a direct comparison of compound activity. To enable direct comparison with the asexual activities the gametocyte numbers used in the assays were adjusted to be similar to the asexual image based assay. Ultimately, a panel of assays, directly comparing asexual, early and late stage gametocyte activity in comparative conditions has been established. The data for in-plate controls, signal to background and Z’ demonstrate the stability of the gametocyte assays over 25 individual 384-well assay plates screened as a single screening campaign. This is equivalent to 9,600 data points per assay. Not only has activity for a range of current anti-malarials and activity for unknowns been identified, the suitability of these gametocyte imaging assays to be performed in true HTS mode has been demonstrated. It is believed this is the first HTS screening data presented for comparison of activity against early stage (I-III) and, of particular importance, late stage (IV-V) gametocytes.

## Competing interests

The authors declare that they have no competing interests.

## Authors’ contributions

SD designed and performed the experimental research, SD analysed data and wrote the paper, VMA designed the research, analysed data and contributed to writing the paper. Both authors read and approved the final manuscript.

## Supplementary Material

Additional file 1 **Early (I-III), late (IV-V) gametocyte screening results for MMV malaria box and corresponding compound data provided by MMV.** The table provides the HTS two dose screening results and subsequent IC_50_ values for actives plus the compound information provided by MMV. Click here for file
